# Clinician Attitudes and Experiences in Screening Patients for Social Determinants of Health Using PRAPARE

**DOI:** 10.13023/jah.0604.04

**Published:** 2025-01-29

**Authors:** Pranav Rane, Mathew B. Weimer, Adam Baus

**Affiliations:** West Virginia University; Valley Health Systems, Huntington, West Virginia; West Virginia University

**Keywords:** Appalachia, PRAPARE, Primary Care, Screening, Social Determinants of Health

## Abstract

**Introduction:**

Social Determinants of Health (SDOH) are the conditions in which people are born, grow, live, work, and age. SDOH significantly influence health outcomes; as such, healthcare systems should screen for patients’ social needs. PRAPARE is standardized screening tool designed to assess and address patients’ social needs.

**Purpose:**

This study examines SDOH screening at Valley Health Systems, a federally qualified health center based in Huntington, West Virginia. The aim of this research is to assess clinician attitudes and experiences in using the PRAPARE tool to screen for SDOH.

**Methods:**

A self-administered online survey was conducted from April to May 2022 among Valley Health Systems clinicians. The survey focused on SDOH screening frequency, clinician attitudes, barriers to screening, and PRAPARE tool usage. Survey questions were adapted from previously published instrument.

**Results:**

Among the 36 clinicians (response rate: 24.0%) who participated, 55.6% sometimes, rarely, or never asked about patient social needs and 47.2% sometimes, rarely, or never reviewed patient charts for social needs information. Common barriers to screening included patient discomfort (38.9%), lack of referral systems (30.6%), and time constraints (27.8%). Although only 30.6% used the PRAPARE tool, those who did found it easy to use (81.8%) and helpful in identifying social needs (81.8%). However, 63.6% felt inadequately trained in using the tool.

**Implications:**

Similar to national trends, some clinicians at Valley Health Systems are overall supportive of SDOH screening. However, they face many common barriers to screening despite integration of PRAPARE. The results of this study provide valuable insights for those navigating the complexities of implementing SDOH screening tools and sustaining use within clinical settings.

## INTRODUCTION

The World Health Organization defines social determinants of health (SDOH) as the conditions in which people are born, grow, live, work, and age.^1^ Public health in the United States (U.S.) is increasing efforts to account for SDOH in its approaches to understanding and addressing healthcare needs and inequities.^2^ Similarly, healthcare systems are increasing efforts to address SDOH among patients and families served – particularly through systematic SDOH screening. Over the past few years, screening tools and processes have been developed to aid clinicians in identifying the social needs of their patients, such as safe housing, healthy foods, and transportation. These screening tools are intended to make it easier for clinicians to screen for both social needs and SDOH. 3 Social needs refer to the individual-level factors that impact a person’s health, while SDOH encompass the population-level conditions that influence health outcomes.^4^

While SDOH screening tools in health care are becoming more common, national research shows that some clinicians are hesitant to screen for social needs for several reasons.^5^,^6^,^7^ Studies indicate that during a routine patient appointment, most clinicians do not have sufficient time to cover both the patients’ medical and social needs.^5^,^6^,^7^ Furthermore, clinicians are at times hesitant to screen patients for social needs because they are uncomfortable in doing so, lack referral systems to help address a patients social needs, and lack training to use the screening tool and act on the results.^5^,^6^,^7^ With clinicians expressing such significant reservations to screening for SDOH, it is critical that we evaluate the effectiveness of SDOH screening tools from a clinician perspective while continuing to assess clinician experiences and attitudes regarding SDOH screening.

Understanding the use of SDOH screening tools is particularly important in Appalachian states like West Virginia (WV), as these communities tend to face unique socioeconomic and health challenges. Appalachia experiences higher rates of poverty, unemployment, morbidity, and mortality as well as lack of access to healthcare compared to the rest of the U.S.^8^ SDOH factors such as food insecurity, low education level, housing instability, and inadequate transportation are also more pronounced in Appalachia, leading to poor health outcomes and large health disparities.^8^,^9^ For clinicians in Appalachia, SDOH screening tools are essential to identifying and addressing the non-medical needs of the individuals within these communities, ultimately helping to improve health outcomes. However, there is a lack of research specifically exploring the use of SDOH screening tools in healthcare systems across Appalachia. This gap highlights the need for targeted studies to assess current screening practices and identify strategies to integrate SDOH screening tools into clinical workflows in this region.

Valley Health Systems, a federally qualified health center (FQHC) headquartered in Huntington, WV, has been at the forefront of integrating SDOH screenings into primary care workflows to better serve their patients in rural areas. In 2016, this health system first incorporated patient screening questions for SDOH in routine clinic visits, and in 2018 implemented the Protocol for Responding to & Assessing Patients’ Assets, Risks & Experiences (PRAPARE).^10^ PRAPARE is a “standardized patient social risk assessment protocol” that helps clinicians identify the social needs of their patients.^11^ It assesses factors outside of medical conditions and care such as housing stability, food security, employment, education, transportation, and social support. PRAPARE is “customizable for various healthcare settings and is frequently integrated into electronic health records (EHRs) to streamline usage.” 10

Between April and May 2022, the West Virginia University Office of Health Services Research partnered with the West Virginia Department of Health’s Division of Health Promotion and Chronic Disease to collect data on PRAPARE. Their findings showed that PRAPRE enhanced SDOH data collection within the health system and helped Valley Health Systems better connect patients to local resources.^10^ However, there is little information on the effectiveness of the PRAPARE tool and its impact on the attitudes and experiences of clinicians who administer the tool and use the data collected. The purpose of this study is to assess the attitudes and experiences of screening patients for SDOH and using PRAPARE among clinicians within Valley Health Systems. It is hypothesized that clinicians in the Valley Health Systems system who use PRAPARE are more likely to screen their patients for SDOH, are more comfortable discussing SDOH with their patients, and are more successful in identifying patient social needs.

## METHODS

This study utilized a self-administered online survey via Qualtrics survey software. The survey was designed to collect descriptive information focused on identifying frequency of SDOH screening, clinician attitudes towards SDOH screenings and its impact on patient health, the barriers clinicians may face when completing SDOH screenings, clinician experiences using PRAPARE, and ability to apply the information it collects. Survey questions were adapted from a priorly published instrument that examined clinician experiences and attitudes towards screening for SDOH within a large health system^4^ and further developed to focus on the current SDOH screening environment at this health system.

Clinicians employed by Valley Health Systems were the target population for this study. The sampling frame consisted of clinicians from the health system who have direct contact with and provide care to Valley Health patients. Examples include physicians (MD or DO), advanced practice providers (PA or NP), nurses (MSN, BSN, RN, or LPN), social workers (LICSW or LGSW), dentists (DDS or DMD), optometrists, dental hygienists, case managers, and others. Clinicians were selected for study given their interactions with patients and experience in incorporating SDOH screenings and use of PRAPARE.

The survey was launched on Monday, April 11, 2022, and closed on May 8, 2022. Valley Health System’s Chief Medical Officer (CMO) distributed the survey through the health systems email. The initial email contained information about the purpose of the study, Institutional Review Board (IRB) approval details, a cover letter, and a voluntary survey link allowing the clinician to participate. The CMO also sent two reminder emails reiterating the study’s purpose, providing the survey link, and noting the study close date. Participation in the survey was completely voluntary and no compensation was provided to the participants or Valley Health System’s CMO for distributing the survey.

### Ethics Approval

This study received IRB approval from the West Virginia University Office of Human Research Protections, Protocol #2202533255.

## RESULTS

All analyses were conducted in SAS JMP® Pro 16.0.0. Data were analyzed using categorical data techniques and descriptive statistics such as frequency distributions. While a total of 42 clinicians completed the survey, six responses were excluded due to partial survey completion. The final study sample was 36 clinicians, representing a response rate of 24.0% (36/150). Demographic analysis indicates that the majority of the participants were nurse practitioners (33.3%), physicians (19.4%), other (19.4%), and dentists (16.7%). The participants that marked “other” included dental hygienists, medical assistants, and counselors. The majority of the participants had been working in the healthcare system for 0 to 5 years (58.3%) or 6 to 10 years (27.8%), were female (66.7%), were between the ages of 41 to 50 (36.1%) or 31 to 40 (27.8%), and white (86.1%) (Table 1).

When asked about how often clinicians addressed the social needs of their patients, majority of clinicians (55.5%) responded that they sometimes (33.3%), rarely (19.4%), or never (2.8%) asked about their patient’s social needs, and 47.2% responded that they sometimes (33.3%), rarely (11.1%), or never (2.8%) review their patient’s chart for information regarding their patients’ social needs. However, of those physicians who did ask about social needs or reviewed patient charts for social needs information (52.8%), 77.8% of the clinicians stated that they always (30.6%) or often (47.2%) use patients’ information about social needs to make medical decisions or develop a care plan.

When asked about the barriers that clinicians face in addressing patient social needs, the most common barriers reported were (1) concerns about patients feeling uncomfortable about answering questions about social needs (38.9%); (2) lack of an established referral system to community organizations to help address patient’s needs (30.6%); and (3) lack of time to discuss patients’ social needs (27.8%) **(**Table 2). Even though clinicians reported facing significant barriers in addressing patient social needs, the majority of clinicians agreed that information about patients’ social needs could be used to improve patient care (91.7%); improve communication with patients (100.0%); and improve trust with patients (94.5%) . The majority also responded that they supported efforts to incorporate social needs into health care (88.9%) and that screening for social needs should be a standard part of care (80.6%) **(**Table 2). When asked who should be screened for social needs, most clinicians (44.6%) responded that all members of the patient population should be screened for social needs followed by members with complex conditions (20.0%) and members with one or more chronic conditions.

**Figure 1:**
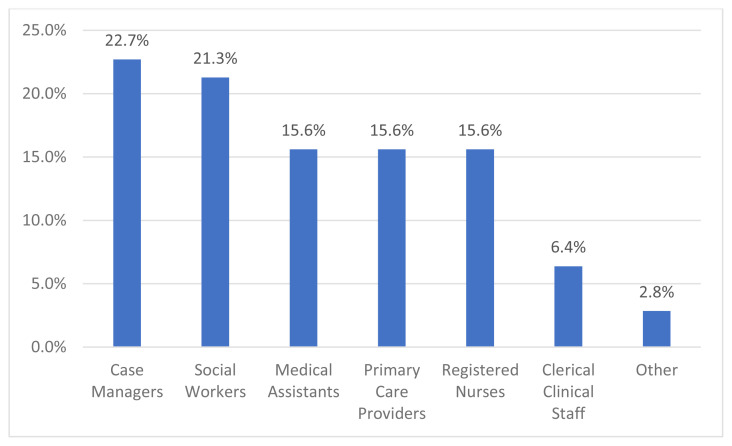
Who should be responsible for screening patients for social needs?

In response to being asked if clinicians used the PRAPARE screening tool to assess patient social needs, only 30.6% reported using the tool. However, of the 30.6% of clinicians that did use PRAPARE, the majority strongly agreed or agreed that the tool (1) was easy to use (81.8%); (2) helped better identify the social needs of the patient (81.8%); (3) increased clinician confidence in asking patients about their social needs (81.8%); (4) and the tool helped the clinician connect patients to community resources (72.7%) (Table 3). The majority of clinicians (63.6%) reported feeling neutral or disagreed about having been adequately trained in using PRAPARE. Clinicians were also asked, based on their own level of expertise and considering the resource and time constraints of SDOH screening, which clinical departments or roles they felt were best suited to screen patients for their social needs. The majority of clinicians stated case managers (22.7%) or social workers (21.3%) were best suited to screen patients. (Fig 1).

## IMPLICATIONS

Although the PRAPARE screening tool was implemented at Valley Health Systems in 2016, 55.6% of the clinicians report sometimes, rarely, or never screening their patients. This finding suggests that not all clinicians use PRAPARE, which is corroborated by the fact that only 30.6% of the respondents reported having used PRAPARE. The low response rate as well as the integration of the tool into the EMR without clear denotation of its name or purpose may bias the reported use. However, of the 30.6% that do use PRAPARE, the majority strongly agree or agree that the screening tool is easy to use (81.8%) and helpful in identifying patient social needs (81.8%). One negative finding about the screening tool is that 63.6% of clinicians feel neutral or disagreed about having been adequately trained in using PRAPARE. Lack of training can be another reason that clinicians at this health system are not using the screening tool on a regular basis.

The most common barriers reported by the clinicians in this study sample are concerns about patients feeling uncomfortable answering questions about social needs (38.9%), lack of an established referral system to community organizations to help address patient’s needs (30.6%), and lack of time to discuss patients’ social needs (27.8%). These three reasons match what previous research found to be potential barriers when assessing patient attitudes and experiences with screening for SDOH.^4^ Finally, even though PRAPRE is available for use to all clinicians, most of the surveyed clinicians responded that case managers (22.7%) or social workers (21.3%) are best suited to screen patients for social needs – providing another reason why uptake of PRAPARE might be lower than anticipated.

Generally, some clinicians at the Valley Health System are supportive of SDOH screening, yet they face substantial barriers to doing so – even with the implementation of PRAPARE. Similar to national trends, the results of this study demonstrate substantial challenges in institutionalizing a SDOH screening tool like PRAPARE and provide insights that may be useful to other FQHCs working to screen for and address SDOH during routine office visits. The public health field must continue to ensure that not only are we screening patients for social needs within healthcare settings, but that we also adequately support clinicians by providing them with easy-to-use screening tools, streamlined processes, training, and resources to support patient needs. This is especially important in the Appalachian Region, which has poorer health outcomes compared to the rest of the country^8^; the need for clinicians to effectively identify and address a patient’s social need is extremely high.

This study has several limitations to consider when interpreting the findings. First, the survey is voluntary and anonymous, which means that the researchers could not collect data on non-respondents. This limitation makes it difficult to assess differences between respondents and non-respondents which restricts the ability to generalize the findings, as non-respondents may have different experiences and/or perspectives from respondents. Additionally, the anonymity of the survey introduced another limitation: the inability to follow up with practitioners who expressed interest in utilizing PRAPARE, which may have limited insights into their experiences or the barriers and facilitators regarding its adoption.

Another limitation of the study is that it used a pre-established instrument that focused on commonly recognized barriers to SDOH screening. By taking this approach, the researchers might have overlooked other factors and/or barriers that could influence use of the screening tool. Finally, this is an exploratory case study focused on gaining an initial understanding of clinician experiences and challenges with SDOH screening at Valley Health Systems at a specific point in time, which limits the generalizability of results. However, future studies should build on the findings of this study by incorporating larger sample sizes and qualitative methods like interview or focus groups, which could yield deeper insights into clinician experiences. Future studies should also explore additional barriers to better understand adoption of screening tools like PRAPARE. While this small sample size and self-reported data of this study limit statistical significance and generalizability, the findings align with national trends and can be used for future research into SDOH screening efforts in other healthcare settings.

**SUMMARY BOX**

**What is already known about this topic?**

Prior research has established the critical role of Social Determinants of Health (SDOH) in influencing health outcomes, prompting healthcare systems to integrate systematic SDOH screening tools like PRAPARE. Existing knowledge also recognizes clinician hesitancy and common barriers, such as time constraints and discomfort, in conducting SDOH screenings, despite the increasing awareness of the importance of addressing social factors in patient care.

**What is added by this report?**

This report contributes new insights by specifically examining clinician attitudes and experiences with SDOH screening, focusing on the implementation of the PRAPARE tool at Valley Health Systems. It sheds light on the challenges faced by clinicians in a real-world setting, offering valuable lessons for improving the effectiveness and integration of SDOH screening tools in healthcare.

**What are the implications for future research?**

The study suggests a need for future research to address barriers hindering the effective implementation of SDOH screening tools, emphasizing the importance of tailored interventions and comprehensive clinician training. Additionally, exploring strategies for seamless integration of tools like PRAPARE into healthcare workflows is crucial for optimizing the effectiveness of SDOH screening initiatives.

## Figures and Tables

**Table 1:** Participant Characteristics (N = 36)

		Frequency	%
**Current Role**			
	Dentist	6	16.7
	Nurse Practitioner	12	33.3
	Physician	7	19.4
	Physician Assistant	1	2.8
	Psychologist	2	5.6
	Registered Nurse	1	2.8
	Other	7	19.4
**Years of Experience**			
	0 – 5 years	21	58.3
	6 – 10 years	10	27.8
	11 – 15 years	1	2.8
	15+ years	4	11.1
**Gender**			
	Women	24	66.7
	Men	12	33.3
**Age**			
	18 – 30	2	5.5
	31 – 40	10	27.8
	41 – 50	13	36.1
	51 – 60	7	19.4
	61 – 70	2	5.6
	71+	1	2.8
	Prefer not to respond	1	2.8
**Race/Ethnicity**			
	Asian or Pacific Islander	1	2.8
	Black	1	2.8
	White	31	86.1
	Hispanic or Latino	0	0
	Native American or Alaskan Native	0	0
	Prefer not to respond	3	8.3

**Table 2:** Addressing Patient Social Needs: A Clinician Perspective

Barriers to Social Needs Screening					
	Strongly Agree	Agree	Neutral	Disagree	Strongly Disagree
I am concerned that patients will feel uncomfortable answering questions about their social needs.	2.8%	44.4%	13.9%	33.3%	5.6%
There is an established referral system to local organizations that can address social needs once they are identified.	11.1%	36.1%	22.2%	27.8%	2.8%
I have adequate time to discuss social needs with my patients.	8.3%	33.3%	30.6%	22.2%	5.6%
I am confident in my ability to help patients address their social needs.	13.9%	38.9%	25.0%	22.2%	0.0%
I am aware of resources available to address patients' social needs.	13.9%	22.2%	41.7%	22.2%	0.0%
I have adequate training/education on how to discuss social needs with my patients.	11.1%	47.2%	25.0%	16.7%	0.0%
I have adequate training/education on how to respond to social needs once they are identified	8.3%	47.2%	22.2%	16.7%	5.6%
I am comfortable asking my patients about their social needs.	33.3%	41.7%	11.1%	13.9%	0.0%
I have support staff to help in talking with my patients about social needs.	16.7%	50.0%	16.7%	13.9%	2.7%
I have adequate Electronic Health Record integration or clinical decision support	13.9%	30.6%	38.9%	11.1%	5.5%
**Clinician Attitudes Regarding Screening for and Addressing Social Needs**					
Information about patients' social needs could be used to improve patient care.	58.3%	33.3%	8.4%	0.0%	0.0%
Information about patients' social needs could be used to improve communication with patients.	47.2%	52.8%	0.0%	0.0%	0.0%
Information about patients' social needs could be used to improve trust with patients.	41.7%	52.8%	5.6%	0.0%	0.0%
I support efforts to incorporate social needs into health care.	47.2%	41.7%	5.6%	5.6%	0.0%
Screening for social needs among patients should be a standard part of care.	41.7%	38.9%	11.1%	8.3%	0.0%

**Table 3:** Clinician Experience with PRAPARE Tool

	Strongly Agree	Agree	Neutral	Disagree	Strongly Disagree
The PRAPARE screening tool was easy to use.	9.1%	72.7%	18.2%	0.0%	0.0%
The PRAPARE screening tool helped me better identify the social needs of my patients.	9.1%	72.7%	18.2%	0.0%	0.0%
The PRAPARE screening tool increased my confidence in asking patients about their social needs.	9.1%	72.7%	18.2%	0.0%	0.0%
The PRAPARE screening tool helped me connect my patients with community resources.	9.1%	63.6%	27.3%	0.0%	0.0%
The PRAPARE screening tool helped my medical decision making by identifying my patients’ social needs.	9.1%	54.6%	36.6%	0.0%	0.0%
I was adequately trained in using the PRAPARE tool.	9.1%	27.7%	54.6%	9.1%	0.0%
The PRAPRE tool is effective in identifying the social needs of my patients.	9.1%	45.5%	36.4%	9.1%	0.0%
